# Development of an integrated CRISPRi targeting ΔNp63 for treatment of squamous cell carcinoma

**DOI:** 10.18632/oncotarget.25678

**Published:** 2018-06-26

**Authors:** Masakazu Yoshida, Etsuko Yokota, Tetsushi Sakuma, Tomoki Yamatsuji, Nagio Takigawa, Toshikazu Ushijima, Takashi Yamamoto, Takuya Fukazawa, Yoshio Naomoto

**Affiliations:** ^1^ Department of General Surgery, Kawasaki Medical School, Okayama, 700-8505 Japan; ^2^ Department of General Internal Medicine 4, Kawasaki Medical School, Okayama, 700-8505 Japan; ^3^ Department of Mathematical and Life Sciences, Graduate School of Science, Hiroshima University, Hiroshima, 739-8526 Japan; ^4^ Division of Epigenomics, National Cancer Center Research Institute, Tsukiji, Chuo-ku, Tokyo, 104-0045 Japan

**Keywords:** CRISPRi, ΔNp63, squamous cell carcinoma, molecular targeted therapy

## Abstract

*TP63* encodes TAp63, which is functionally similar to the tumor suppressor *TP53*, and ΔNp63, which lacks the transcription-activating domain of TAp63 and appears potently oncogenic in squamous cell carcinomas (SCCs). In this study, we developed an integrated CRISPR interference (CRISPRi) system to selectively suppress ΔNp63 (CRISPRiΔNp63). We engineered this CRISPRi using tandemized guide RNA expression cassettes that targeted the 50 to 100 bp downstream of the transcription start site of ΔNp63 in combination with inactivated Cas9 linked to the transcription repression module Krüppel-associated box repressor domain. The plasmid vector harboring CRISPRiΔNp63 repressed *ΔNp63* transcription in lung and esophageal SCC cells. Likewise, Ad-CRISPRiΔNp63, an all-in-one adenoviral vector containing the tandemized gRNAs and dCas9/KRAB expression cassette suppressed ΔNp63 expression in SCC cells. Ad-CRISPRiΔNp63 also effectively decreased cell proliferation and colony formation and induced apoptosis in lung and esophageal SCC cells *in vitro* and significantly inhibited tumor growth in a mouse lung SCC xenograft model *in vivo*. These results indicate that ΔNp63 suppression using CRISPRiΔNp63 may be an effective strategy for treating lung and esophageal SCC.

## INTRODUCTION

The development of gene editing methods using engineered zinc finger nucleases (ZFNs) and transcription activator-like effector nucleases (TALENs) enabled precise genetic modification through induction of targeted DNA double-strand breaks [1, 2]. However, the difficulty of creating the ZFN or TALEN constructs was a limitation of using these chimeric nucleases to facilitate gene targeting. A more powerful tool now available is the CRISPR/Cas9 system, a RNA-guided site-specific DNA cleavage technology based on an immune mechanism that protects bacteria from foreign DNA. The most widely used version is CRISPR/Cas9 from *Streptococcus pyogenes* [3], which has two important advantages over ZFNs and TALENs. First, the design and construction of a CRISPR/Cas9 system is easier than systems using ZFNs and TALENs. Second, by introducing multiple gRNAs, CRISPR/Cas9 can be used to target several genes or sequences at the same time [4]. In addition, catalytically inactive Cas9 (dCas9) fusion protein guides that use gRNAs have been developed to target selected DNA sequences to inhibit (CRISPRi) or activate (CRISPRa) transcription of target genes [5].

With 1.6 million deaths annually, lung cancer is the most frequent cause of cancer-related deaths worldwide, causing [6]. However, recent progress in next-generation sequencing has enabled non-small cell lung cancer (NSCLC) patients with specific genomic alterations to benefit from molecular targeted therapies. Indeed, up to 69% of patients with advanced NSCLC could have a potentially actionable molecular target [7]. Molecularly targeted drugs for EGFR mutation or ALK fusion genes have led to remarkable improvement in personal therapies, especially in pulmonary adenocarcinoma. Several driver mutation candidates have also been identified in lung squamous cell carcinoma (SCC), though effective targeted therapies have not yet been established. Similarly, the 5-year survival rate for patients with esophageal SCC remains poor [8, 9] due in large part to a lack of effective treatment strategies.

*TP63* is a member of the *TP5*3 tumor suppressor family and encodes multiple isoforms of p63. Its two promoters (P1 and P2) mediate generation of two classes of proteins: TAp63, which contains an N-terminal transactivation (TA) domain, and ΔNp63, an N-terminal truncated isoform lacking the TA domain of TAp63 [10]. As a result of gene amplification, ΔNp63α is the more abundantly detected isoform in human squamous cell carcinomas, including those in the lung [11, 12], and silencing ΔNp63 using siRNA suppresses growth of ΔNp63-expressing cancer cells [13–15]. Conversely, overexpression of ΔNp63 in normal keratinocytes and fibroblasts increased colony growth in soft agar and xenograft tumor formation in nude mice [13, 16]. Thus, ΔNp63 appears to be oncogenic.

To make the most of CRISPR/Cas9, we previously established a system for construction of all-in-one expression vectors containing multiple gRNA expression cassettes and a Cas9 nuclease expression cassette and demonstrated efficient targeting for multiplex genome editing [17]. In the present study, we developed a vector containing expression cassettes with multiple gRNAs targeting ΔNp63 with dCas9 fused to the KRAB (dCas9/KRAB) expression cassette [18, 19]. We then investigated whether this integrated CRISPRi system targeting ΔNp63 would exert an antitumor effect in lung and esophageal SCC *in vitro* and *in vivo*.

## RESULTS

### Detection of ΔNp63 expression in lung and esophageal cancer cells and normal cells

**Figure 1:**
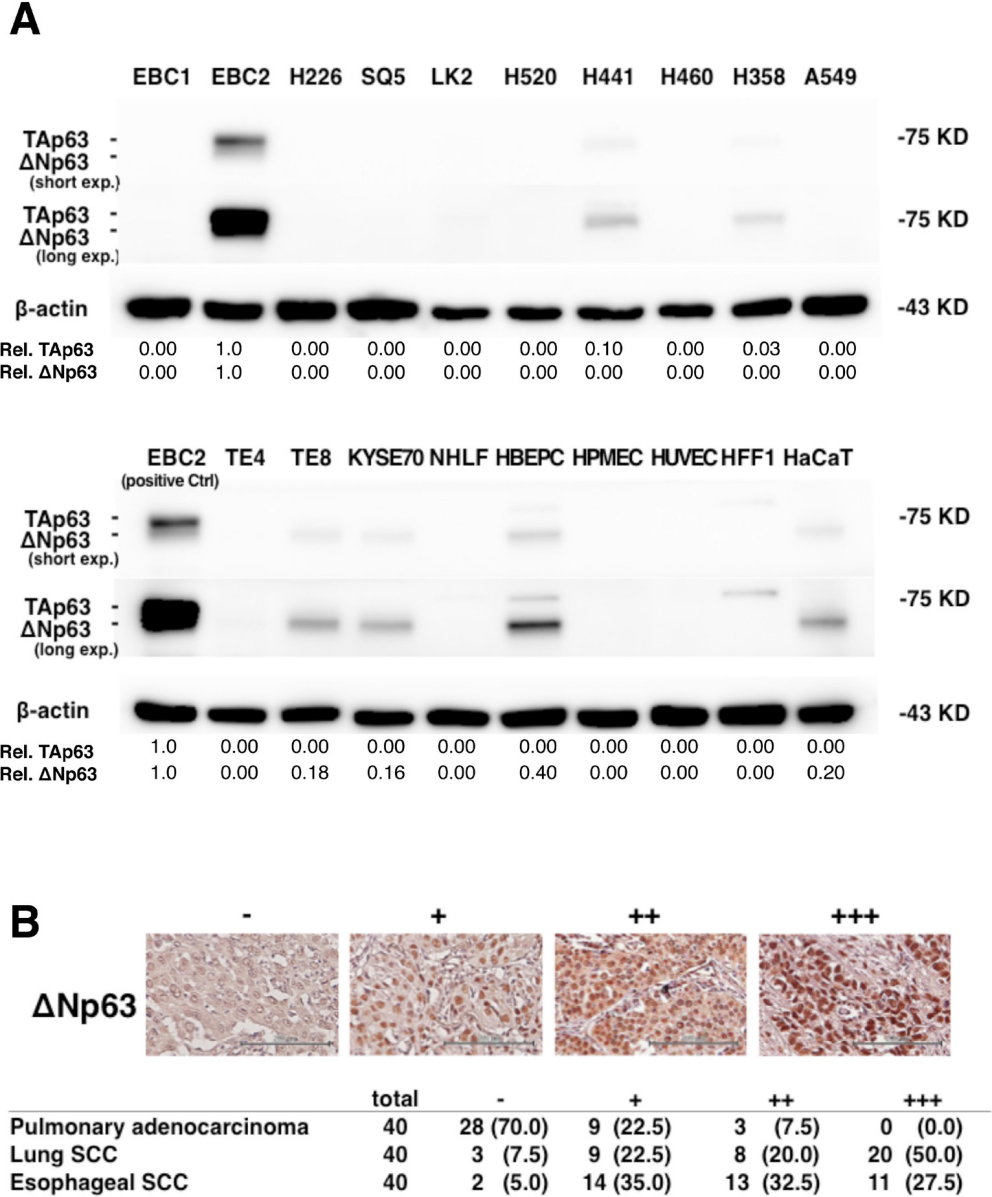
ΔNp63 expression in lung and esophageal SCC and pulmonary adenocarcinoma (**A**) Immunoblot analysis of TAp63 and ΔNp63 expression in the indicated cells. β-actin served as a control. A non-specific band with higher molecular weight than TAp63 (75 kDa) was seen in HBEPCs and HFF1s. Band densities normalized to the EBC2 cell band are shown below the blots. (**B**) Representative immunohistochemistry results. ΔNp63 staining intensity was scored as follows: none = −, weak = +, moderate = ++, and strong = +++ expression. Scale bars = 200 μm. Shown below are the frequencies of ΔNp63 expression in samples of primary pulmonary adenocarcinoma, lung SCC, and esophageal SCC. Percentages are given in parentheses.

We initially performed immunoblot assays to assess ΔNp63 expression in lung and esophageal cancer cells and normal cells. As shown in Figure 1A, ΔNp63 expression was detected in EBC2 lung SCC cells and in TE8 and KYSE70 esophageal SCC cells, as well as in HBEPCs and HaCaT cells. By contrast, no expression was detected in any of the pulmonary adenocarcinoma cell lines test (H441, H460, H358, A549) or in NHLFs, HPMECs, HUVECs or HFF1s. TAp63 expression was detected in EBC2, H441 and H358 cells. But whereas only three of the nine human SCC cell lines tested expressed ΔNp63 (EBC2, TE8 and KYSE70) [20, 21], ΔNp63 expression was detected in more than 92.5% of human lung and esophageal SCC specimens (Figure 1B). In addition, ΔNp63 expression was detected in 30% of pulmonary adenocarcinomas. These results suggest ΔNp63 is commonly expressed in human lung and esophageal SCC and that molecular targeting of ΔNp63 may be a useful approach to treating SCC.

### The integrated CRISPRi system suppressed ΔNp63 transcriptional activity in lung and esophageal SCC cells

**Figure 2:**
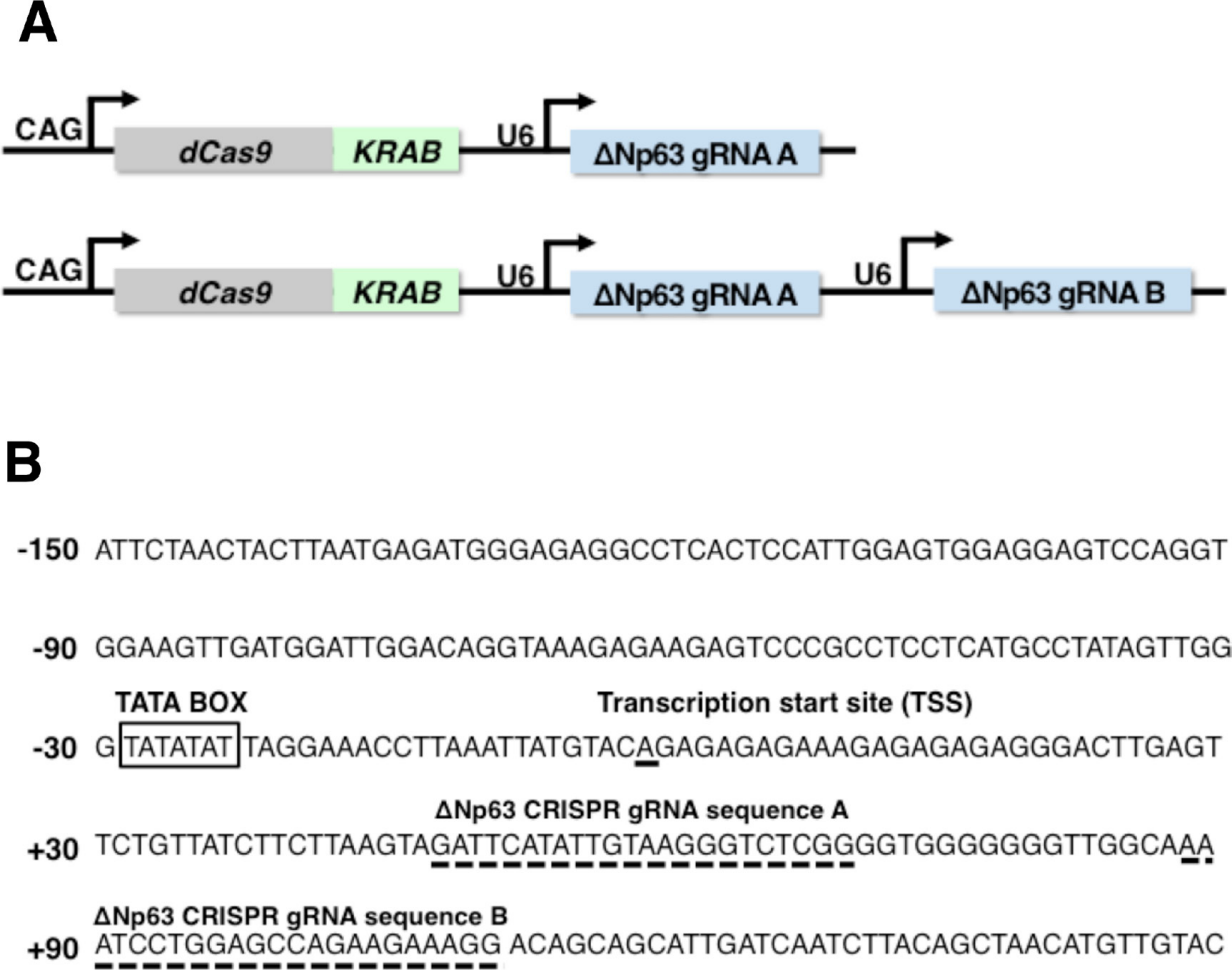
Multiplex gene targeting using dCas9/KRAB and tandemized gRNA expression cassettes (**A**) Schematic representation of an integrated CRISPRi system encoding single or double gRNAs targeting different genomic loci and a dCas9/KRAB fusion protein. These dCas9/KRAB and gRNAs expression cassettes were ligated into a single construct (pCRISPRiΔNp63A and pCRISPRiΔNp63A/B, respectively). (**B**) Sequence of the ΔNp63 proximal promoter. Broken lines indicate the gRNA targeting sequences. A TATA box and the transcription start site (TSS) are also indicated.

**Figure 3:**
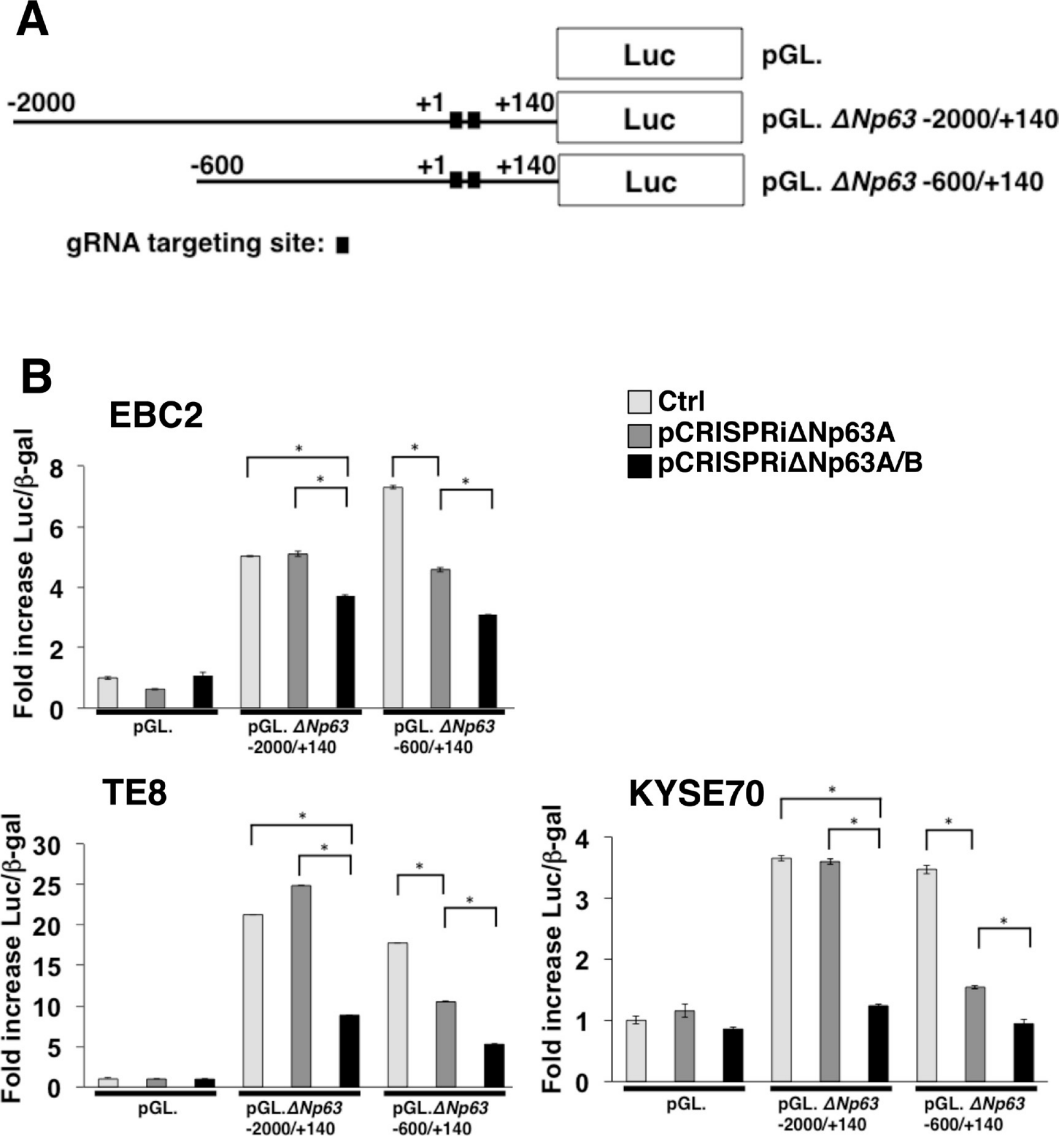
Repression of ΔNp63 promoter activity using a CRISPRi system targeting ΔNp63 in lung and esophageal SCC cells (**A**) Schematic representation of Δ*Np63* distal and proximal promoter reporter constructs. Luc; Luciferase. (**B**) Transient transfection reporter assays in EBC2 lung SCC cells and TE8 and KYSE70 esophageal SCC cells using the indicated Δ*Np63* luciferase reporter constructs (2 µg, pGL) with dCas9/KRAB + gRNA expression constructs (pCRISPRiΔNp63A or pCRISPRiΔNp63A/B, 2 µg) and pCMV. β-gal (1 µg). pX330A_dCas9/KRAB-1x2 vector, not expressing gene-specific gRNA but expressing dCas9/KRAB, was used as control (Ctrl). Results are presented as fold induction of relative light units normalized to β-galactosidase activity relative to that observed with control constructs. Bars represent the mean ± SD (*n* = 3). ^*^p < 0.01. Each experiment was repeated at least three times, and supplementary data sets are shown in Supplementary Figure 1.

To suppress ΔNp63 expression in lung and esophageal SCC, we constructed all-in-one expression vectors containing single or double gRNA expression cassettes complementary to target sequences in the ΔNp63 promoter combined with dCas9/KRAB expression cassettes (pCRISPRiΔNp63A and pCRISPRiΔNp63A/B, respectively) (Figure 2A and 2B). To test the efficacy of these vectors, we measured ΔNp63 transcriptional activity in lung and esophageal SCC cells with and without expression of gRNAs complementary to the target sequences in the ΔNp63 promoter along with dCas9/KRAB expression. In the controls, which lacked gRNA expression, the promoter regions −2000/+140 and −600/+140, which contain the gRNA targeted sites, exhibited significant increases in transcriptional activity in EBC2 lung SCC cells (5.03-fold and 7.30-fold, respectively), in TE8 esophageal SCC cells (24.9-fold and 17.8-fold, respectively), and in KYSE70 esophageal SCC cells (3.65-fold and 3.47-fold, respectively) 24 h after transfection (Figure 3A and 3B). On the other hand, pCRISPRiΔNp63A and pCRISPRiΔNp63A/B significantly decreased transcriptional activity in ΔNp63 promoter region −600/+140 in EBC2 cells (4.35-fold and 3.08-fold, respectively), TE8 cells (10.5-fold and 5.28-fold, respectively), and KYSE70 cells (1.94-fold and 0.95-fold, respectively). pCRISPRiΔNp63A/B also significantly suppressed transcriptional activity of the ΔNp63 promoter region −2000/+140 in EBC2 cells (3.70-fold), TE8 cells (8.85-fold) and KYSE70 cells (1.23-fold). These results indicate that pCRISPRiΔNp63A/B more effectively suppressed ΔNp63 transcriptional activity than pCRISPRiΔNp63A in these SCC cell lines (Figure 3A, 3B and Supplementary Figure 1). Likewise, pCRISPRiΔNp63A/B inhibited expression of ΔNp63 protein more effectively than did pCRISPRiΔNp63A in EBC2 and TE8 cells (Supplementary Figure 2). Thus the integrated CRISPRi system targeting ΔNp63 significantly suppresses ΔNp63 expression in lung and esophageal SCC cells, and using double gRNAs that target different promoter regions in the same gene at the same time suppresses the transcriptional activity and protein expression more effectively than using a single gRNA.

### Ad-CRISPRiΔNp63 suppresses ΔNp63 expression in SCC cells and immortalized keratinocytes

**Figure 4:**
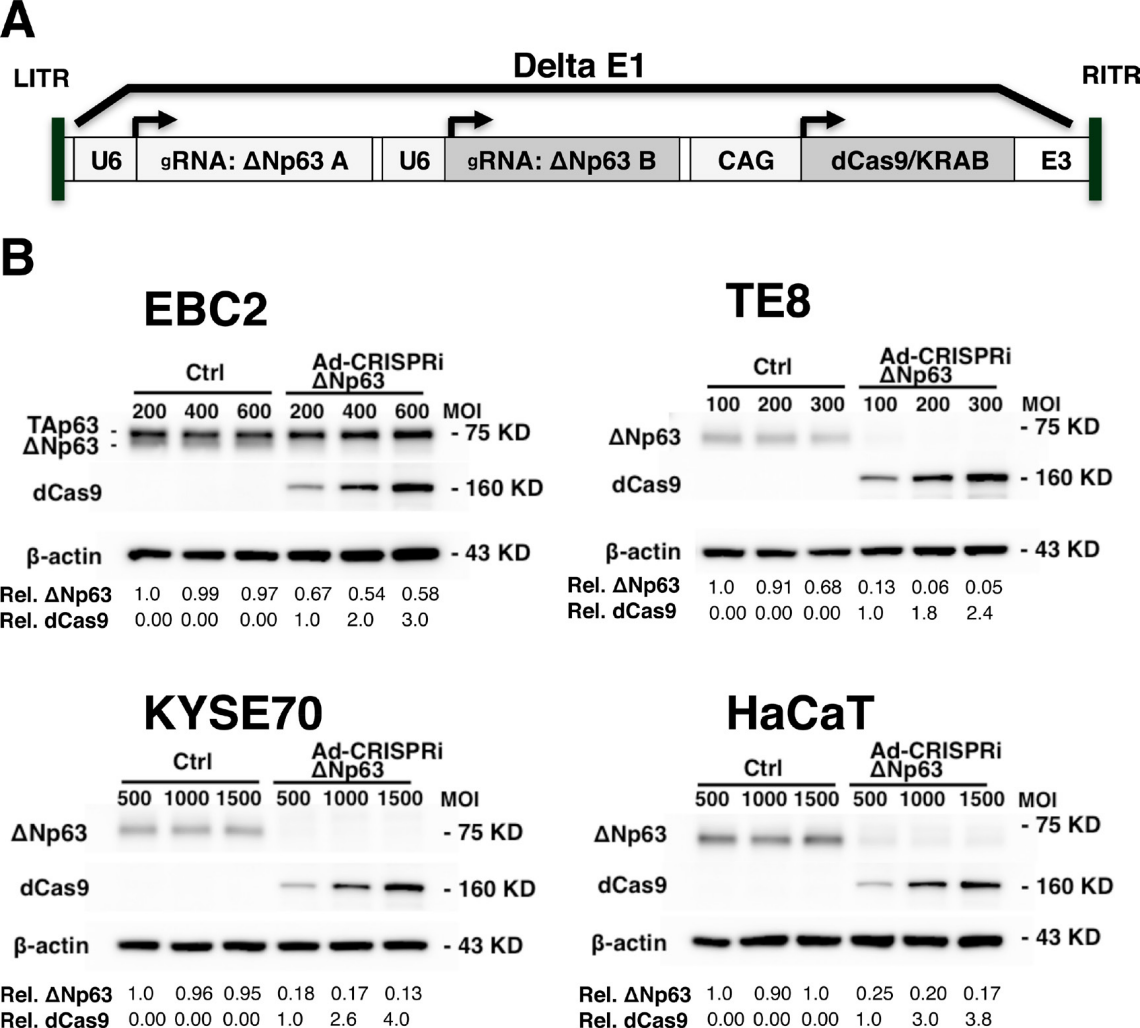
Ad-CRISPRiΔNp63 suppressed ΔNp63 expression in lung and esophageal SCC cells and in immortalized keratinocytes (**A**) Schematic representation of the all-in-one adenoviral vector Ad-CRISPRiΔNp63, which encodes double gRNAs with dCas9/KRAB. (**B**) Immunoblot analysis showing Ad-CRISPRiΔNp63 suppresses ΔNp63 expression in EBC2 lung SCC cells, KYSE70 and TE8 esophageal SCC cells, and HaCaT immortalized keratinocytes 48 h after adenoviral infection. In EBC2 cells, both TAp63 (upper band) and ΔNp63 (lower band) were detected. Relative band densities are shown below the blots. For ΔNp63, band densities normalized to Ad-empty administered at the lowest MOI. For dCas9, band densities are normalized to Ad-CRISPRiΔNp63 administered at the lowest MOI.

We next evaluated whether ΔNp63 expression could be suppressed in SCC cells and keratinocytes using an all-in-one adenoviral vector containing double gRNA expression cassettes and a dCas9/KRAB expression cassette to target ΔNp63 (Ad-CRISPRiΔNp63) (Figure 4A). We found that Ad-CRISPRiΔNp63 dose dependently increased dCas9 expression in EBC2 lung SCC cells, TE8 and KYSE70 esophageal SCC cells, and HaCaT immortalized keratinocytes 48 h after infection. Moreover, Ad-CRISPRiΔNp63 clearly suppressed ΔNp63 expression in all tested cell lines. On the other hand, the control vector did not effectively inhibit ΔNp63 expression in the cells (Figure 4B).

### Ad-CRISPRiΔNp63 suppresses colony formation and induces apoptosis in ∆Np63expressing SCC cells and immortalized keratinocytes

**Figure 5:**
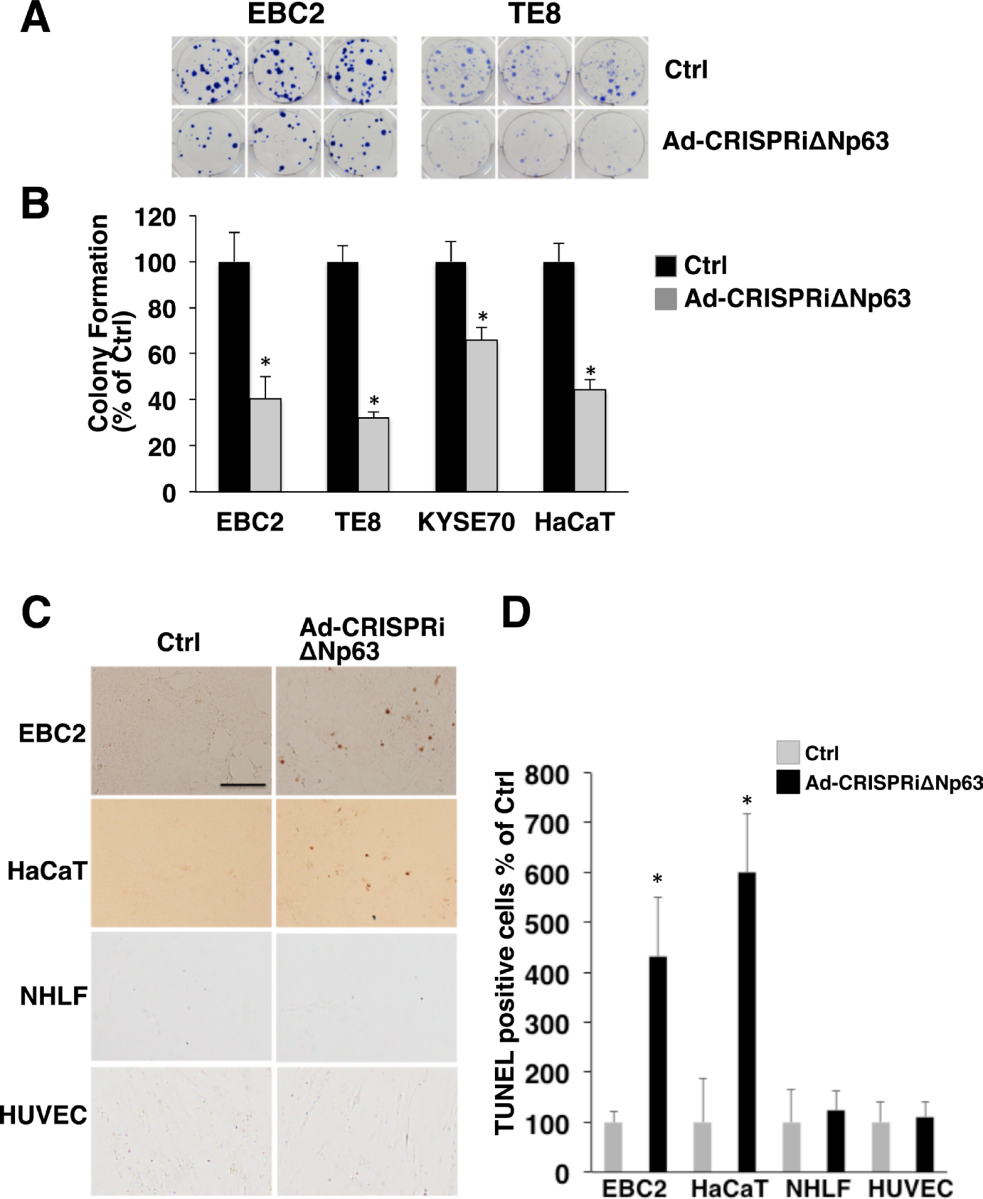
Ad-CRISPRiΔNp63 significantly decreases colony formation and induces apoptosis in ΔNp63-expressing SCC cells and keratinocytes (**A**) Colony formation by EBC2 lung SCC cells, TE8 and KYSE70 esophageal SCC cells, and HaCaT cells treated with Adempty (Ctrl) or Ad-CRISPRiΔNp63. Shown are representative images of experiments performed in triplicate with EBC2 and TE8 cells. (**B**) Mean colony numbers derived from triplicate dishes for each treatment. Counts obtained in the control condition (Ctrl) were arbitrarily set to 100%, and Ad-CRISPRiΔNp63 is shown relative to Ctrl. Bars depict the mean ± SD (*n* = 3). ^*^p < 0.01. (**C**) Ad-CRISPRiΔNp63 increases the incidence of TUNEL-positive cells among EBC2 and HaCaT cells but not NHLFs or HUVECs 48 h after infection. Adenoviral vectors were administered at a MOI of 600 for EBC2 cells, 1500 for HaCaT cells, and 50 for NHLFs and HUVECs. Shown are representative images of experiments. Scale bar = 200 µm. (**D**) Mean TUNEL-positive cells/per field. Bars depict the mean ± SD (*n* = 5). Data are shown relative to the control group. ^*^p < 0.01.

To elucidate the antitumor effect of the Ad-CRISPRiΔNp63 system, we assessed colony formation by in SCC cells and keratinocytes after Ad-CRISPRiΔNp63 infection. As shown in Figure 5A and 5B, Ad-CRISPRiΔNp63 significantly decreased colony formation by EBC2, TE8, KYSE70, and HaCaT cells. TUNEL assays revealed that the incidence of apoptosis was increased in EBC2 and HaCaT cells 48 h after Ad-CRISPRiΔNp63 infection. By contrast, little or no apoptosis was seen in NHLFs and HUVECs after Ad-CRISPRiΔNp63 infection (Figure 5C, 5D and Supplementary Figure 3). Hoechst staining also showed the presence of apoptotic cells among EBC2 cells after Ad-CRISPRiΔNp63 infection, but not among NHLFs (Supplementary Figure 4). These results indicate that Ad-CRISPRiΔNp63 exerts an antitumor effect against ΔNp63-positive SCC cells and immortalized keratinocytes, but not normal fibroblasts or endothelial cells.

### Down-regulation of Δ*Np63* suppresses lung SCC growth in a xenograft mouse model

**Figure 6:**
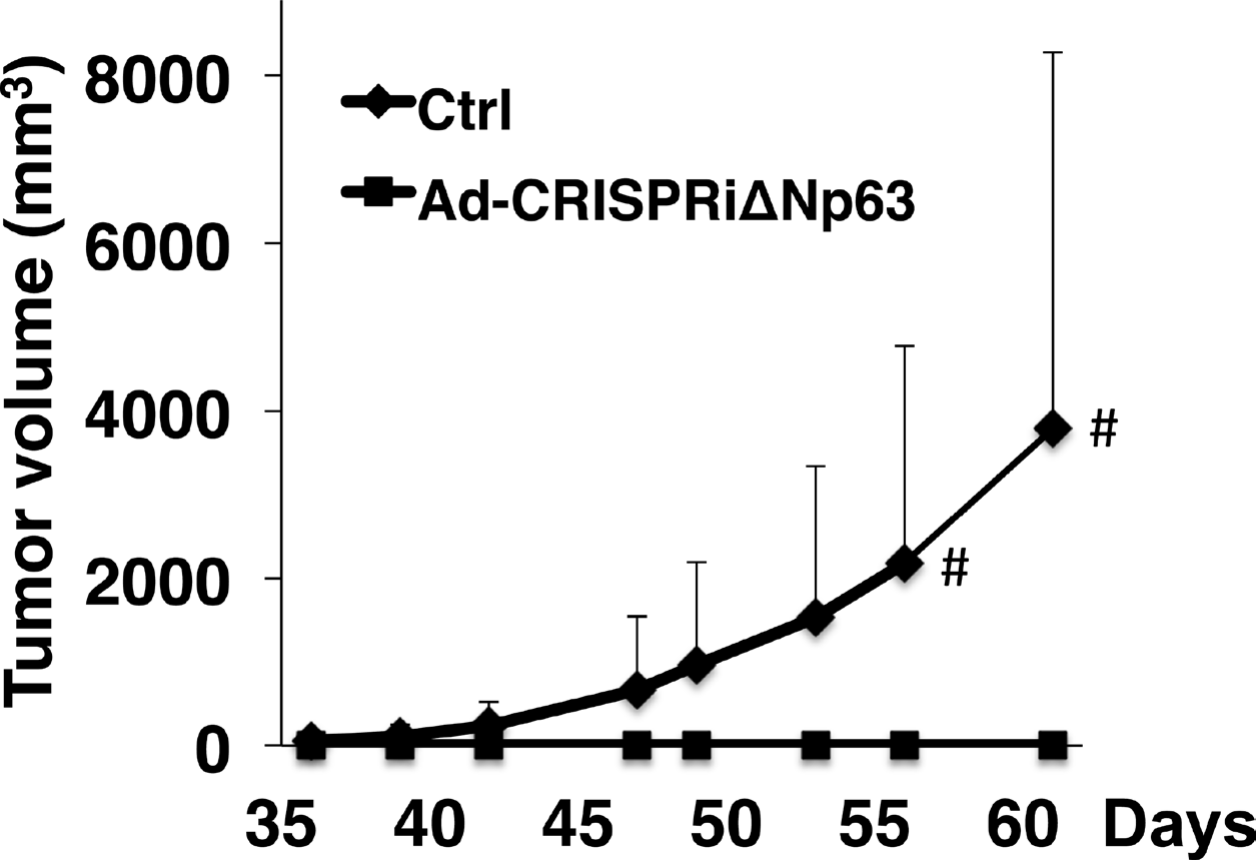
Ad-CRISPRiΔNp63 significantly reduces lung SCC growth in a mouse xenograft model Shown are volumes of tumors derived from EBC2 lung SCC cells treated with Ad-empty (Ctrl) or Ad-CRISPRiΔNp63. Prior to the inoculations, cells were infected with adenoviral vectors at a MOI of 300. Symbols depict the mean tumor volume + SD (*n* = 10 in each group). ^#^*p* < 0.05. In the control group, xenograft tumors developed in 5 of 10 mice but in none of the 10 mice in the Ad-CRISPRiΔNp63 group (*p* = 0.0389; Fisher’s exact test).

In addition to the cell-based experiments, we used an EBC2 lung SCC xenograft tumor model in nude mice to determine whether CRISPRiΔNp63 suppresses tumor growth *in vivo*. We found that tumor growth was significantly suppressed after Ad-CRISPRiΔNp63 infection at a MOI of 300 (Figure 6) or 600 (Supplementary Figure 5). These results indicate that Ad-CRISPRiΔNp63 exerts a marked antitumor effect against lung SCC *in vivo*.

## DISCUSSION

Although use of RNAi may be the simplest approach to knocking down expression of a target gene [22], it sometimes exhibits marked off-target effects and unpredictable knockdown efficiencies [23, 24]. CRISPRi provides more consistent and robust gene knockdown in mammalian cells [25]. However, previous studies have shown that CRISPR/Cas9 can frequently cause off-target mutagenesis [26, 27], though several strategies have been developed to reduce off-target effects of CRISPRi [28, 29]. In addition, ChIP sequencing showed that dCas9 binds to a number of off-target sites and most dCas9-gRNA complexes are able to bind to non-target sites [30, 31]. Apparently, there are numerous potential non-specific binding sites for Cas9 and dCas9. It is noteworthy, however, that CRISPRi is strongly dependent on the genomic loci of dCas9 binding. Down-regulation of gene expression requires dCas9/KRAB to be targeted to a narrow window of DNA extending from 50 to 100 bp downstream of the transcription start site (TSS) [25]. It would therefore be expected that off-targeted binding of dCas9/KRAB to sites other than that small window would not induce transcriptional repression. These results indicate that CRISPRi using dCas9/KRAB and gRNAs is much safer than a Cas9-based gene knockout system or RNAi, which both exhibit greater off-target effects.

The main practical advantage of the CRISPR/Cas9- or CRISPR-based approach (e.g., CRISPR i/a), as compared to ZFNs or TALENs, is the ease of multiplexing [4]. The simultaneous binding of Cas9 or the dCas9/KRAB-gRNA complex at multiple genomic loci could enable one to edit, repress or activate several genes simultaneously. In the present study, the single or double gRNA expression cassettes complementary to the target sequences in the ΔNp63 promoter effectively suppressed expression of ΔNp63 mRNA and protein in the presence of dCas9/KRAB. Moreover, double gRNA expression systems simultaneously targeting different promoter regions in the same gene more effectively suppress transcriptional activity than using a single gRNA.

Recently, Hussein *et al.* use transcriptomes derived from epidermal cells from TAp63−/− and ΔNp63−/− mice to conduct pan-cancer analysis of The Cancer Genome Atlas (TCGA) to identify transcriptional networks regulated by TAp63 and ΔNp63 [32]. They identified lymphoid enhancer-binding factor 1 (LEF1) as a major downstream target of ΔNp63 that plays a crucial role in cancer development, and the activity of ΔNp63 was pleiotropic in various kinds of cancer, suggesting LEF1 could be another useful target for the treatment of certain cancers, including SCC. On the other hand, TNF-α, Ras and TGF-β may work as upstream regulatory signals of ΔNp63 that stimulate cancer progression [33, 34].

*TP63* is also known to be a critical transcription factor in some organs [35]. On the other hand, ΔNp63 is reportedly the major isoform in human lung, where it is expressed exclusively in epithelial basal cells [36]. In the present study, ΔNp63 was detected not only in cancer cells and immortalized keratinocytes but also in normal basal cells from human bronchial epithelium. This indicates CRISPRiΔNp63 may injure normal cells that express ΔNp63 (Figure 1A, Supplementary Figure 6). For clinical use, therefore, we plan to locally inject Ad-CRISPRiΔNp63 into lung and esophageal SCC using bronchoscopy and digestive endoscopy, to minimize normal tissue and cell damage which might occur with systemic injection. Importantly, Purushothama *et al.* observed that when basal cells are injured, luminal secretory cells can dedifferentiate into basal cells to compensate for the loss of basal cellular function [37].

Viral vectors are the most effective means of introducing genetic material into various kinds of cells *in vitro* and *in vivo*. Adenoviral vectors are non-enveloped, non-integrating double stranded DNA vectors that enter cells mainly via coxsackie-adenovirus receptors. These vectors can effectively deliver genes into a broad range of cell types, whether dividing and non-dividing. This is unlike single stranded RNA retroviral vectors, which require dividing cells. Both adenoviral and adeno-associated viral vectors are considered safe because they do not integrate into the host chromosome and pose no risk of genotoxicity or insertional mutagenesis [38, 39]. Importantly, adeno-associated viral vectors are classified as non-pathogenetic and can be used under the lowest biosafety standards in any laboratory. However, these vectors have a limited packaging capacity (up to 4.7 kb) [40]. Adenoviral vectors have a larger cargo capacity (up to 35 kb) [41], which enabled us to construct the all-in-one adenoviral vector used in this study. The advantage of all-in-one vectors is that dCas9 and gRNAs are consistently delivered to, and expressed in, the same cell in a fixed ratio [17, 42].

In this study we used integrated adenoviral CRISPRi technology to successfully induce an anti-tumor effect in lung and esophageal SCCs. One major disadvantage of adenoviral vectors relates to their ability to induce inflammation [43, 44], which compromises their efficacy and safety in clinical trials. Recently, however, it was reported that gutless adenoviral vectors constructed by eliminating all residual viral genes stimulated less T-cell immune activity [45, 46]. In addition to the usage of these kinds of vectors, cancer and/or tissuespecific promoter systems and tumor-selective replication competency should result in safer and more effective therapies based on adenovirally mediated CRISPRi system that are useful for effective treatment of lung and esophageal SCC [47, 48].

## MATERIALS AND METHODS

### Cell lines and culture conditions

H520 and H226 human lung SCC cells; H358, H441, H460 and A549 human pulmonary adenocarcinoma cells; TE4 and TE8 human esophageal SCC cells; and HFF1 human foreskin fibroblasts were obtained from the American Type Culture Collection (Manassas, VA, USA) and grown in RPMI 1640 (H226, H358, H460, TE4, TE8) or high-glucose Dulbecco’s modified Eagle medium (DMEM) (H520, H441, A549, HFF1) supplemented with 10% heat-inactivated fetal bovine serum (FBS). KYSE70 human esophageal SCC cells were obtained from the Japanese Collection of Research Bioresources (Tokyo, Japan) and grown in RPMI 1640 supplemented with 10% heat-inactivated FBS. EBC1, EBC2, SQ5 and LK2 lung SCC cells were kindly provided by Dr. Katsuyuki Kiura (Department of Respiratory medicine, Okayama University Graduate School of Medicine and Dentistry, Okayama, Japan) and grown in RPMI 1640 supplemented with 10% heat-inactivated FBS. HaCaT spontaneously immortalized keratinocytes were a kind gift from Dr. Yumi Aoyama (Department of Dermatology, Kawasaki Medical School) and were grown in high glucose DMEM supplemented with 10% heat-inactivated FBS. Human umbilical vein endothelial cells (HUVECs) were purchased from Thermo Fisher Scientific (Rockford, IL, USA) and grown in Endothelial Cells Growth Medium (Medium 200) supplemented with Low Serum Growth Supplement using a kit from Thermo Fisher Scientific. Normal human lung fibroblasts (NHLFs) were obtained from Clonetics (San Diego, CA, USA) and grown in high-glucose DMEM with 10% heat-inactivated FBS. All cell lines were cultured in 5% CO_2_ at 37° C. Cell pellets of human bronchial epithelial cells (HBEPCs) and human pulmonary microvascular endothelial cells (HPMECs) were purchased from PromoCell (Heidelberg, Germany).

### Construction of pCRISPRiΔNp63A and pCRISPRiΔNp63A/B plasmid vectors

The pCRISPRiΔNp63A and pCRISPRiΔNp63A/B plasmids were constructed using a Multiplex CRISPR/Cas9 Assembly System Kit (Addgene, Cambridge, MA, USA; Kit #1000000055) and a Multiplex CRISPR dCas9/FokI-dCas9 Accessory Pack (Addgene, Kit # 1000000062) as previously described [17, 49] with some modification. Briefly, KRAB domain was added to the pX330A_dCas9-1x2 vector contained in the Accessory Pack using PCR amplification and an In Fusion HD Cloning Kit (Takara Bio Inc., Otsu, Japan), yielding pX330A_dCas9/KRAB-1x2. The oligonucleotides for the template of gRNAs targeting ΔNp63 were then annealed and inserted into pX330A_dCas9/KRAB-1x2 and into pX330S-2, which was contained in the Assembly System Kit, to create pCRISPRiΔNp63A and pCRISPRiΔNp63B, respectively. To construct pCRISPRiΔNp63A/B, pCRISPRiΔNp63A and pCRISPRiΔNp63B were assembled using the Golden Gate cloning method as described previously [17]. The sequences of the oligonucleotides for the gRNA templates were as follows: ΔNp63A_s, CACCGATTCATATTGTAAGGGTCT; ΔNp63A_as, AAACAGACCCTTACAATATGAATC; ΔNp63B_s, CACCGAAATCCTGGAGCCAGAAGAA; and ΔNp63B_as, AAACTTCTTCTGGCTCCAGGATTTC.

### Luciferase reporter construct and transient transfection reporter assay

ΔNp63 promoter from human genomic DNA was obtained from Invitrogen Life Technologies (Carlsbad, CA, USA). The position of the transcription start site (+1) for ΔNp63 was determined using the Ensembl Human Genome browser. The luciferase reporter constructs pGL.ΔNp63 2000/+140 and pGL.ΔNp63 −600/+140 were generated by subcloning the ΔNp63 promoter region −2000/+140 or −600/+140 amplified from genomic DNA using PCR primers (5’-ctcggcggcc^HindIII^aagctt^-2000^agtggatatcaatacttggg or 5’-ctcggcggcc^HindIII^aagctt^-600^catgctcgaaaaaatcaggt and 5’-tctagtgtct^HindIII^aagctt^+140^gttagctgtaagattgatcaa) and subcloned into HindIII digested pGL4.23 using an In-Fusion HD Cloning Kit (Takara Bio Inc.). One day before transfection, the cells were seeded into 6-well plates to a density of 2 × 10^5^ per well. Transfections were carried out using Lipofectamine 3000 (Thermo Fisher Scientific) according to the manufacturer’s protocol. Transfected cells were harvested after an additional 24 h. Results of one representative experiment are presented as fold induction of relative light units normalized to β-galactosidase activity relative to that observed with the control vectors [42]. Each experiment was repeated at least three times. Error bars indicate the SD from the average of the triplicate samples in one experiment.

### Adenoviral vectors

Ad-CRISPRiΔNp63 was generated by subcloning the gRNA expression cassettes and the dCas9/KRAB expression cassette from pCRISPRiΔNp63A/B amplified using PCR primers (5′- gtaactataacggtc ctttttacggttcctggcctttt and 5′- attacctctttctccgctccccagcatgcctgctattct) and subcloned into linearized pAdenoX vector using Adeno-X Adenoviral System 3 Universal according to manufacturer’s protocol (Takara Bio Inc.). The viral titer for each vector was determined using an Adeno-X^™^ Rapid Titer Kit (Takara Bio Inc.), and the optimal multiplicity of infection (MOI) was determined by infecting each cell line with Ad-CMV/*GFP* and assessing expression of GFP [50].

### Colony formation assay

Cells were first plated at a density of 2 × 10^5^ cells per well in 6-well plates 24 h before virus infection. The following day, Ad-empty and Ad-CRISPRiΔNp63 were used at a MOI of 600 to infect EBC2 cells, at a MOI of 300 to infect TE8 cells, and at a MOI of 1500 to infect KYSE70 and HaCaT cells. After incubation for 24 h, the cells were harvested by incubation with trypsin/EDTA and counted. EBC2 and KYSE70 cells were then plated in triplicate at a density of 5 × 10^2^ cells per well in 6-well plates, while TE8 and HaCaT cells were plated in triplicate at a density of 1 × 10^3^ cells per well. After incubation for 14 days, the cells were fixed, stained with Diff-Quik (Sysmex, Kobe, Japan) [51], and colonies (groups of at least 50 aggregated cells) were counted. The mean number of colonies in the control group was arbitrarily set to 100%, and all other counts were normalized to the control, and percent specific cytotoxicity towards colony formation was calculated.

### TUNEL staining

Terminal deoxynucleotidyltransferase-mediated dUTP-biotin nick end labeling (TUNEL) was performed to detect apoptosis using the DeadEnd Colorimetric TUNEL System (Promega, Madison, WI, USA) according to the manufacturer’s protocol.

### Immunoblot analysis

Cells were lysed in ice-cold M-PER lysis buffer purchased from Thermo Fisher Scientific. Cell lysates were clarified by centrifugation (20 min at 15,000 rpm and 4° C), and protein concentrations were determined using BCA protein assays (Thermo Fisher Scientific). Equal amounts of protein were separated using SDS-PAGE. The resolved proteins were then transferred to Hybond PVDF transfer membranes (Millipore, Bedford, MT, USA) and incubated with primary and secondary antibodies according to the Supersignal^®^ West Pico chemiluminescence protocol (Thermo Fisher Scientific). Primary anti-TAp63 and anti-ΔNp63 antibodies were obtained from Cell Signaling Technology (Beverly, MA, USA), and an anti-Cas9 antibody was obtained from Novus Biologicals, Inc. (Littleton, CO, USA). An anti-β-actin antibody was obtained from Santa Cruz Biotechnology (Santa Cruz, CA, USA). Secondary horseradish peroxidase (HRP)-conjugated antibodies were from Jackson Immunoresearch Laboratories (West Grove, PA, USA).

### Immunohistochemistry

Sections were deparaffinized through a series of xylene, graded ethanol, and water immersion steps. After autoclaving the sections in target retrieval solution (Dako, Carpinteria, CA, USA; for 15 min, they were incubated with 3% hydrogen peroxide for 5 min to block endogenous peroxidase activity. Specimens were then incubated overnight at 4° C with anti-ΔNp63 antibody (clone 11F12.1, 1:500 dilution; Millipore, Bedford, MA, USA). After three washes with TBS, the sections were treated with streptavidin-biotin complex (Envision System labeled polymer, HRP, Dako, Carpinteria, CA) for 60 min at room temperature. Immunoreactions were visualized using 3,3′-diaminobenzidine (DAB) substrate-chromogen solution (Cytomation Liquid DAB Substrate Chromogen System, Dako) and counterstained with hematoxylin. Sections were then immersed in an ethanol and xylene bath and mounted on slides for examination. For immunohistochemical analysis, 40 lung SCC tissue samples, 40 pulmonary adenocarcinoma tissue samples and 40 esophageal SCC samples in tissue sections were obtained from patients who underwent surgical treatment at Kawasaki Hospital, Okayama, Japan. The experimental protocol was approved by the Ethics Review Committee of Kawasaki Medical School (Ethics Committee reference number: 1310).

### Mouse experiments

The experimental protocol was approved by the Ethics Review Committee for Animal Experimentation of Kawasaki University Graduate School of Medicine and Dentistry (Ethics Committee reference number: 15–046). EBC2 lung SCC cells were plated in 15 cm dishes at a density of 4 × 10^6^ cells per dish, cultured overnight at 37° C, and then infected with Ad-empty or Ad-CRISPRiΔNp63. After incubation for an additional 24 h, the cells were harvested and resuspended in culture medium. Human lung cancer xenografts were established in 6-week-old female BALB/c nude mice (CLEA Japan, Tokyo, Japan) through subcutaneous injection of adenovirus-treated EBC cells (2 × 10^6^ cells/100 µl) into their dorsal flank. The mice were then closely observed, and tumors were measured twice a week. Tumor volumes were calculated using the formula a × b^2^ × 0.5, where a and b are the large and small diameters, respectively.

### Statistical analysis

Differences between the study groups were evaluated using Student’s *t*-test or Fisher’s exact test. Values of *p* < 0.05 were considered significant.

## SUPPLEMENTARY MATERIALS FIGURES


