# Supplementary material for: Development of an integrated CRISPRi targeting ΔNp63 for treatment of squamous cell carcinoma
Source: Oncotarget. 2018 Jun 26;9(49):29220–32. doi: 10.18632/oncotarget.25678 (PMC6044376; doi:10.18632/oncotarget.25678)
Supplement: Supplementary file 1 [file oncotarget-09-29220-s001.pdf]

Development of an integrated CRISPRi targeting ΔNp63 for treatment of squamous cell carcinoma

SUPPLEMENTARY MATERIALS

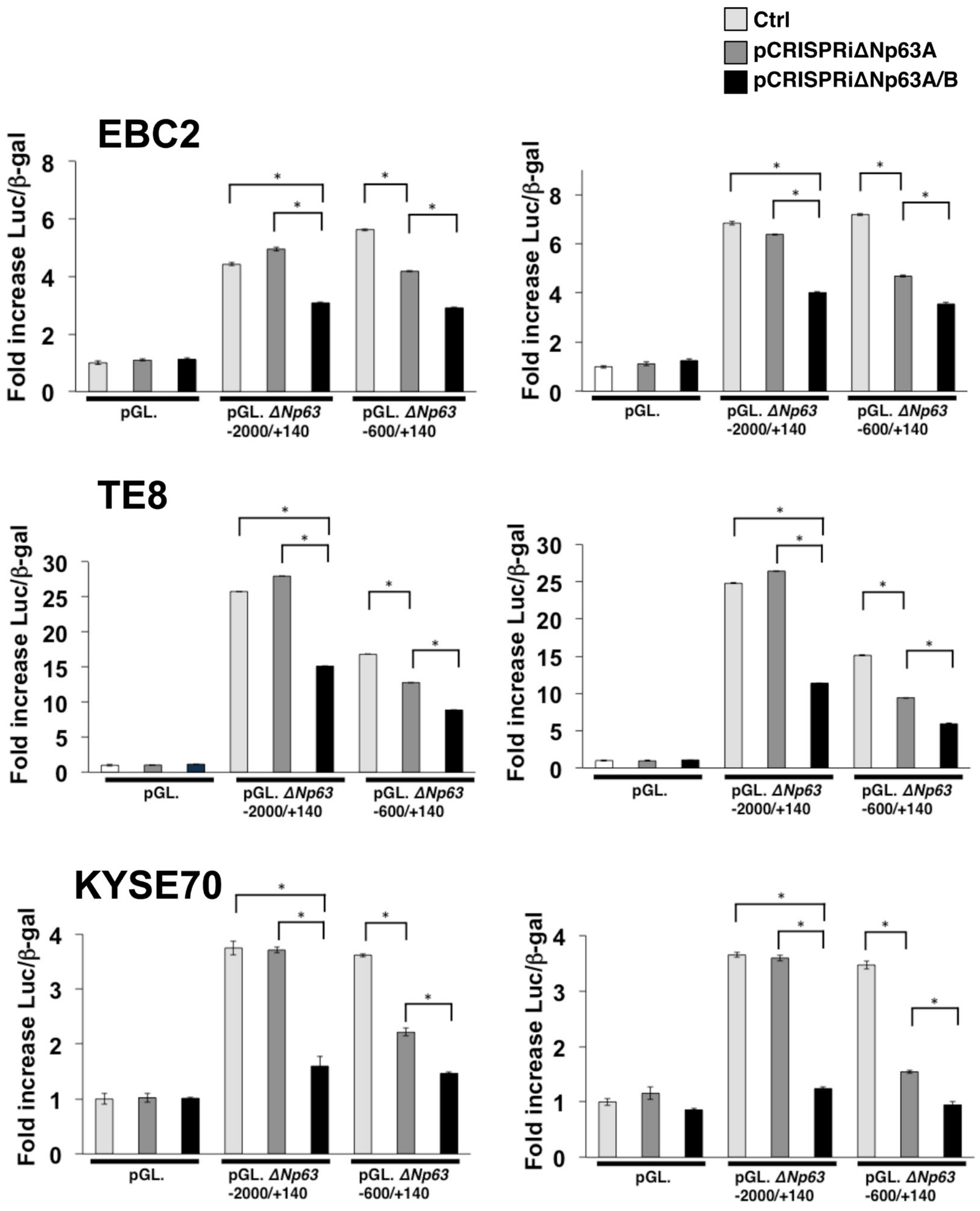

Supplementary Figure 1: Repression of *ΔNp63* promoter activity by the CRISPRi system targeting ΔNp63 in lung and esophageal SCC cells. The other data sets in Figure 3B were shown. Statistical significance was defined as $p < 0.01$ (*).

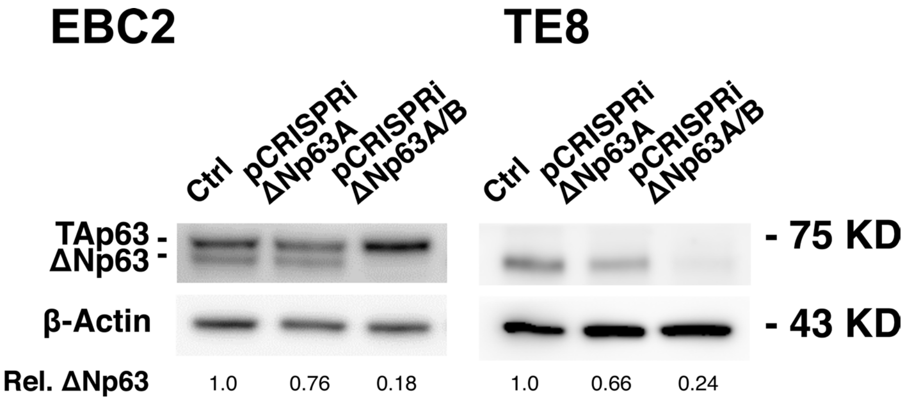

Supplementary Figure 2: pCRISPRiΔNp63A/B effectively suppressed ΔNp63 expression more than pCRISPRiΔNp63A in lung and esophageal SCC cells. Cells were transfected using Lipofectamine 3000 (Thermo Fisher Scientific) according to manufacturer's protocol. Cells were seeded into 6-well plates at a density of 2×10^6 per well for EBC2 cells and 4×10^6 per well for TE8 cells 1 day before transfection. To enrich for transfected cells, a plasmid with the puromycin resistant cassette pPUR (0.5 μg, Takara Bio, Inc.) was co-transfected with pX330A_dCas9/KRAB-1x2 (Ctrl), pCRISPRiΔNp63A or pCRISPRiΔNp63A/B (2 μg). After 24 hours, cells were selected in 2 μg/ml puromycin for 48 hours, then cells were harvested and the protein was isolated according to the manufacturer's instructions. ΔNp63 expressions were quantified using Adobe Photoshop CS5 Extended to analyze optical density probed on the same membrane. Results were presented as intensity of each band compared to intensity of the Ctrl band.

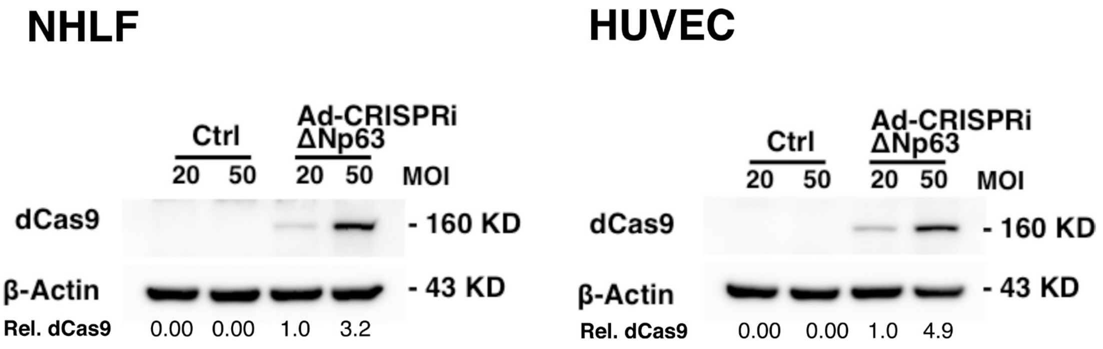

Supplementary Figure 3: Detection of dCas9 in NHLFs and HUVECs after Ad-CRISPRiΔNp63 infection by immunoblot. dCas9 was dose dependently induced by Ad-CRISPRiΔNp63 in NHLFs and HUVECs 48 hours after infection. Ad-empty was used as control (Ctrl). dCas9 expression was quantified using Photoshop CS5 Extended to analyze optical density probed on the same membrane. Results were presented as intensity of each band compared to that of the band of Ad-CRISPRiΔNp63 infected at the lowest MOI.

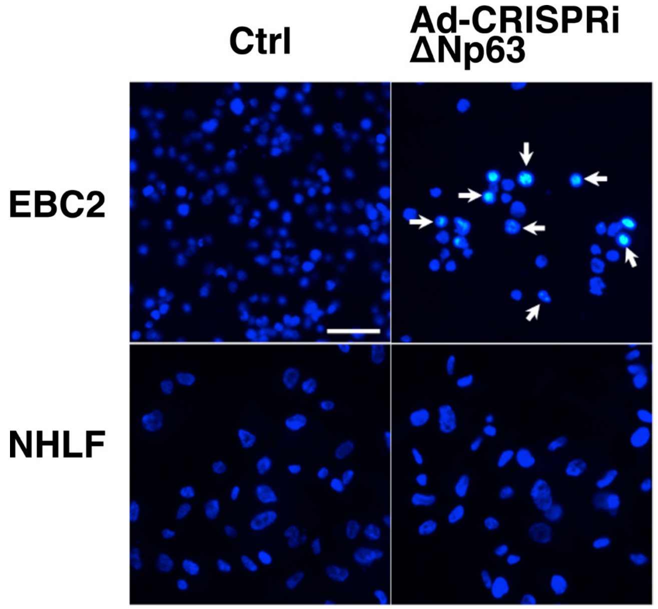

Supplementary Figure 4: Ad-CRISPRiΔNp63 induced apoptosis in EBC2 lung SCC cells but not in NHLFs. Morphologic characteristics of apoptosis were evaluated using Hoechst 33342 dye (Molecular Probes, Eugene, OR), which stains DNA. Cells were incubated with 1 μg/ml Hoechst dye and then visualized under a fluorescence microscope. Ad-CRISPRiΔNp63 increased apoptosis in EBC2 cells (600 MOI) but not in normal NHLFs (300 MOI) 72 hours after infection. Ad-empty was used as control (Ctrl). Apoptotic cells were indicated with arrow. Scale bar shows 50 μm.

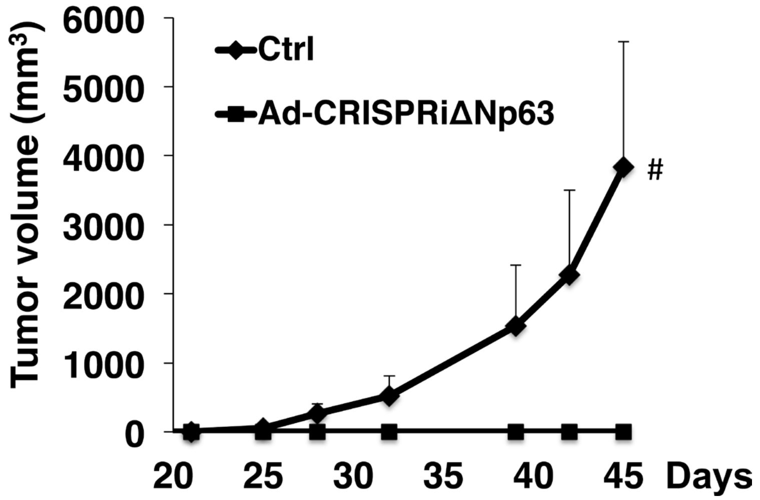

Supplementary Figure 5: Ad-CRISPRiΔNp63 significantly reduced lung SCC growth in xenograft mice model. Volumes of the tumors derived from EBC2 lung SCC cells treated with Ad-empty (Ctrl) or Ad-CRISPRiΔNp63 are shown. Adenoviral vectors were infected at a MOI of 600 for the cells prior to inoculations. The volumes were monitored over time (days) after inoculation of tumor cells. Five mice were studied in each group. Tumor growth is expressed as mean tumor volume; bars represent SD. Statistical significance is assessed by Student's two-sided *t* test compared to the control group. $^{\#}p < 0.05$. In the control group four of five mice formed xenograft tumors (4/5) while none of ten mice formed tumors in Ad-CRISPRiΔNp63 treated group ($p = 0.0476 < 0.05$, Fisher's exact test).

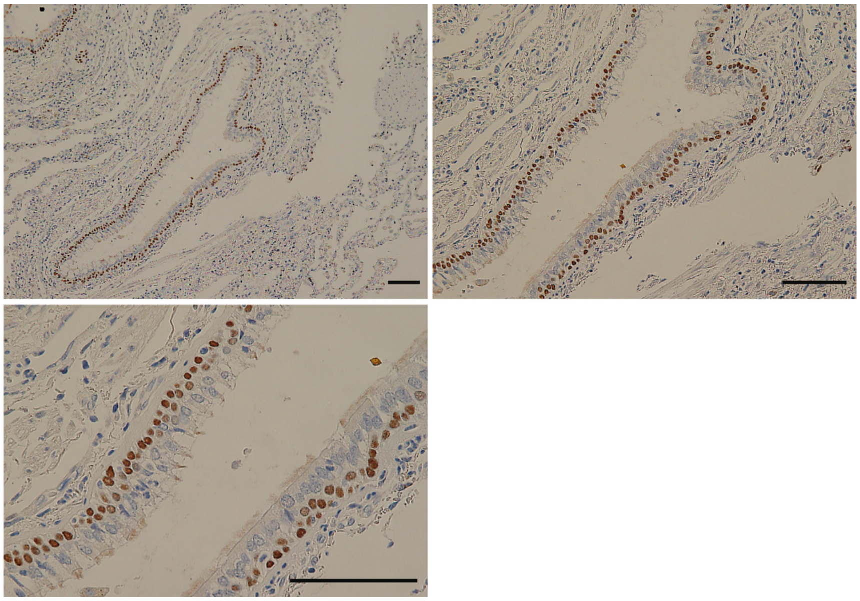

Supplementary Figure 6: ΔNp63 is expressed in basal cells in normal human bronchi. The Staining with the ΔNp63 isoform specific antibody indicates expression in the basal layer of the human bronchial epithelium in the normal part of the specimen. Scale bar shows 100 μm.
